# Contralateral diaphragmatic palsy in acute stroke: An interesting observation

**DOI:** 10.4103/0972-5229.53113

**Published:** 2009

**Authors:** Sudhir Kumar, Rajesh Reddy, Subhashini Prabhakar

**Affiliations:** **From:** Stroke Unit, Institute of Neurological Sciences, Apollo Hospitals, Hyderabad, India

**Keywords:** Acute stroke, clinical implication, diaphragmatic palsy, pathophysiological basis

## Abstract

Diaphragmatic palsy in hemiparetic stroke is not well recognized. Further, its implications on stroke outcome have not been studied. Here, we report a patient with left-sided diaphragmatic palsy due to an acute right middle cerebral artery territory infarction. The diagnosis was suspected on finding an elevated dome of the diaphragm on the left side in a routine chest radiograph and was confirmed by finding decreased movements of the left hemidiaphragm on fluoroscopic examination. We hypothesize that this condition is probably under-recognized in clinical practice and its clinical importance not well known. The pathophysiological basis of diaphragmatic palsy in acute stroke and its possible clinical implications are discussed.

## Introduction

Acute ischemic strokes are commonly encountered in medical practice. The commonest clinical presentation of acute stroke in the middle cerebral artery territory is contralateral hemiplegia. Weakness of upper and lower limbs, facial paresis and gaze palsy are well known clinical findings in hemiplegic stroke. Diaphragmatic palsy in acute hemiplegic stroke is not so well known and there is very little published data.[1–3] We report a case of left hemiplegic stroke associated with diaphragmatic palsy. The pathophysiology of diaphragmatic palsy in acute stroke and its clinical implications are discussed.

## Case Report

A 60-year-old man presented with an acute episode of left hemiparesis of three days duration. Risk factors included diabetes mellitus, hypertension and chronic smoking. On examination, the patient's blood pressure was 140/90mmHg, pulse rate was 90/minute and regular. Carotids were normal. He had a left homonymous hemianopia and a mild left upper motor neuron facial paresis. He had a left hemiparesis with grade 3/5 power (Medical Research Council grading) in left upper and lower limbs.

**Figure 1:**
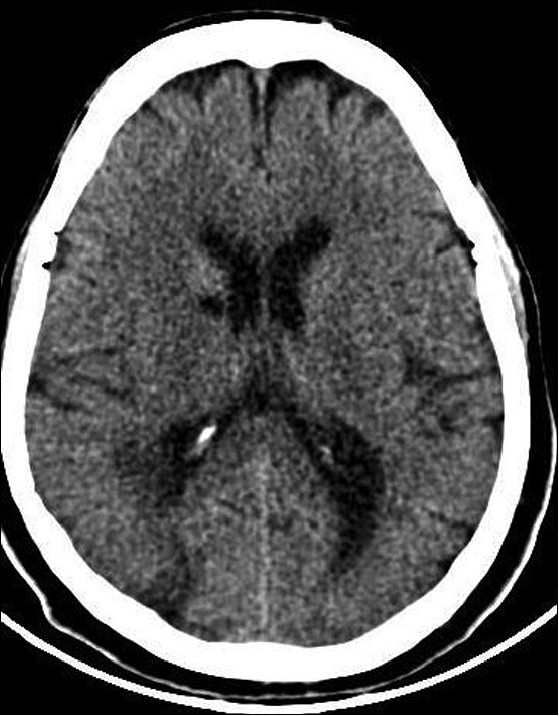
Axial noncontrast computerized tomography of the brain showing infarcts in the right caudate nucleus, internal capsule and parietooccipital cortex

**Figure 2:**
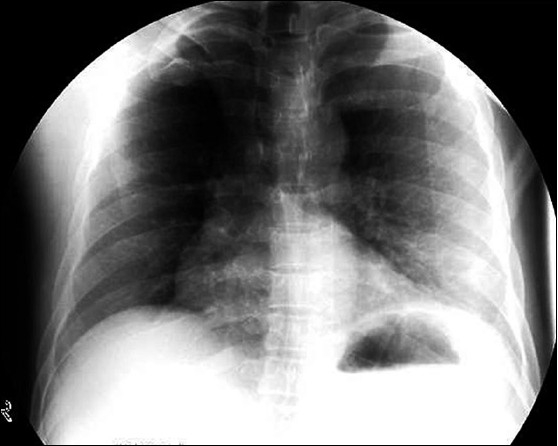
Chest radiograph showing an elevated left hemidiaphragm

Laboratory investigations including hematological and biochemical parameters, were unremarkable. Electrocardiogram, echocardiogram and carotid Doppler studies were normal. Noncontrast CT scan of the brain showed multiple infarcts located in the centrum semiovale, caudate nucleus, genu of internal capsule and parieto-occipital areas on the right side [Figure 1]. A routine chest radiograph showed an elevated left dome of the diaphragm [Figure 2]. A left-sided diaphragmatic palsy was suspected, which was confirmed by fluoroscopic imaging that showed grossly decreased diaphragmatic excursions on the left side. Peripheral nerve conduction study showed a normal compound muscle action potential (CMAP) from the diaphragm on stimulation of the left phrenic nerve that was similar to that of the right side. Diaphragmatic electromyogram (EMG) was also normal. Pulmonary function tests and arterial blood gas analysis were normal.

Electrophysiologic investigations were suggestive of a defect in the central conduction pathway - the corticodiaphragmatic pathway - that was related to the acute ischemic stroke. A final diagnosis of left diaphragmatic palsy with left hemiparesis related to the infarct in the right middle cerebral artery territory was made.

## Discussion

The case described above highlights an unusual complication of stroke - diaphragmatic palsy on the side of hemiparesis.

Other workers have studied the subject of diaphragmatic palsy in stroke earlier. Diaphragmatic elevation in supine chest films in 62 patients with recent stroke was studied by Santamaria *et al*.[1] They found an excessive elevation of the diaphragm on the paretic side in patients with severe hemiparesis as compared to the controls. This finding was seen more commonly with lesions involving the genu of the internal capsule.

Electrophysiological studies have attempted to study the corticodiaphragmatic pathways. Cortical magnetic stimulation was used to study the central conduction times in patients with stroke and healthy volunteers.[2] In this study, left and right conduction times were found to be symmetrical in healthy subjects and patients with hemiplegia but without capsular lesion (16.5 to 20.1ms). Conversely, they were markedly asymmetrical in patients with capsular hemiplegia, diaphragm response being abolished or markedly delayed on the plegic side. This study also provided evidence supporting the presence of a “central diaphragm paralysis” in patients with stroke. In addition, it suggested that there is no bilateral cortical motor representation of each hemidiaphragm.

Corticodiaphragmatic pathways were assessed in another study using magnetic stimulation of the scalp.[3] Decreased diaphragmatic excursion was found in 41% of patients with stroke and abnormal magnetic evoked potentials from the affected hemisphere were found in 70.5% of patients.

An attempt has been made by some workers to localize the diaphragm motor cortical representation using magnetic stimulation in normal adult human subjects.[4,5] The average point of optimal excitability was determined to be 3-4cm lateral to the mid-sagittal plane and 1cm anterior to the pre-auricular plane. These studies also confirmed that both bilateral crossed and uncrossed corticospinal connections to the diaphragm were usually present, with the crossed tract predominating. Ultrasonographic techniques have also confirmed that the diaphragm responds to contralateral and not ipsilateral cortical stimulation.[5]

What are the clinical implications of diaphragmatic palsy in stroke? This question cannot be answered well as there is a lack of studies on this aspect. In one study,[3] a greater degree of respiratory dysfunction including a higher incidence of hypoxia, hypocapnia, and acidosis was seen in patients with hemiplegia and it was significantly related to the diaphragmatic excursion and site of infarction on the CT scan.

Pneumonia is one of the commonest complications occurring in patients with acute stroke and affects about 7% of all patients admitted.[6,7] Mortality is three times higher in patients with pneumonia as compared to those without it.[7] Common factors predisposing patients to pneumonia are altered consciousness, impaired swallowing and cough reflex. We hypothesize that diaphragmatic palsy may also be a contributing factor towards the development of pneumonia as an increased incidence of pneumonia has been observed on the hemiparetic side in patients with stroke.[8] This could be due to a weak cough on the side with diaphragmatic palsy. However, large prospective studies are required to validate this.

In conclusion, diaphragmatic palsy can occur in patients with stroke, especially if it affects the internal capsule. Diaphragmatic palsy predisposes patients to a greater degree of respiratory dysfunction and pneumonia on the hemiparetic side. Further prospective studies on this subject are warranted.
